# Metabolite profiling and biological activities of bioactive compounds produced by *Chrysosporium lobatum* strain BK-3 isolated from Kaziranga National Park, Assam, India

**DOI:** 10.1186/2193-1801-2-122

**Published:** 2013-03-21

**Authors:** C Ganesh Kumar, Poornima Mongolla, Pombala Sujitha, Joveeta Joseph, K Suresh Babu, Gangi Suresh, Kallaganti Venkata Siva Ramakrishna, Uppula Purushotham, G Narahari Sastry, Ahmed Kamal

**Affiliations:** 1 Chemical Biology Laboratory, CSIR-Indian Institute of Chemical Technology, Uppal Road, Hyderabad, Andhra Pradesh 500007 India; 2 Natural Products Chemistry Division, CSIR-Indian Institute of Chemical Technology, Uppal Road, Hyderabad, Andhra Pradesh 500007 India; 3 Nuclear Magnetic Resonance Centre, CSIR-Indian Institute of Chemical Technology, Uppal Road, Hyderabad, Andhra Pradesh 500007 India; 4 Molecular Modelling Group, CSIR-Indian Institute of Chemical Technology, Uppal Road, Hyderabad, Andhra Pradesh 500 007 India

**Keywords:** Acetylcholinesterase inhibitors, *Chrysosporium lobatum*, Curvularin, α, β-Dehydrocurvularin, Molecular docking

## Abstract

In an ongoing survey for bioactive potential of microorganisms from different biosphere zones of India, a new *Chrysosporium lobatum* strain BK-3 was isolated from soil sample collected from a biodiversity hotspot, Kaziranga National Park, Assam, India. Bioactivity-guided purification resulted in the isolation of two bioactive compounds whose chemical structures were elucidated by ^1^H and ^13^C Nuclear Magnetic Resonance (NMR), 2D-NMR, Fourier Transform Infra-red (FT-IR) and mass spectroscopic techniques, and were identified as α, β-dehydrocurvularin and curvularin. Only curvularin exhibited 80% acetylcholinesterase (AChE) inhibitory activity. Detailed ligand receptor binding interactions were studied for curvularin by molecular docking studies. Further, both curvularin and α, β-dehydrocurvularin had similar level of cytotoxicity against different human tumour cell lines like A549, HeLa, MDA-MB-231 and MCF-7, while α, β-dehydrocurvularin was active against COLO 205 with a IC_50_ of 7.9 μM, but curvularin was inactive. α, β-Dehydrocurvularin also showed good superoxide anion scavenging activity with an EC_50_ value of 16.71 μg ml^-1^. Hence, both these compounds exhibited differences in bioactive profiles and this was probably associated with their minor structural differences. This is a first report on bioactive compounds exhibiting AChE inhibitory, cytotoxicity and antioxidant activities from *Chrysosporium lobatum* strain BK-3.

**Electronic supplementary material:**

The online version of this article (doi:10.1186/2193-1801-2-122) contains supplementary material, which is available to authorized users.

## Introduction

Alzheimer’s disease (AD), the most common neurodegenerative disorder associated with ageing, is accompanied by severe deficiency in choline acetyltransferase activity in the hippocampus and cerebral cortex. A significant correlation has been found between the decrease in cortical cholinergic activity and the deterioration of mental test scores in patients suffering from AD. Therefore, potentiation of central cholinergic activity has been proposed as a possible therapeutic approach to improve cognitive function in AD patients ( Johnson et al. 2000 ). Among the most promising strategies is the elevation of the brain concentration of the neurotransmitter acetylcholine by centrally acting acetylcholinesterase (AChE, EC 3.1.1.7) inhibitors. During the past decade, several cholinergic drugs have been launched in the market, including the synthetic compounds like tacrine ( Bartus et al. 1982 ), donepezil (E2020) ( Watkins et al. 1994 ), galanthamine ( Davis et al. 1995 ), and rivastigmine ( Kawakami et al. 1996 ). It has been reported that use of these drugs produce significant cognitive improvement ( Nightingale 1997 ; Kryger et al. 1999 ; McGleenon et al. 1999 ). However, a major drawback in the widespread use of these synthetic compounds as a general therapy is the appearance of undesirable side effects such as hepatotoxicity, which imposes severe dose-limits ( Watkins et al. 1994 ).

The comprehensive study of the AChE/inhibitor complexes by X-ray crystallography had indicated that AChE possessed a narrow gorge with two separate ligand binding sites, an acylation site (active site) and a peripheral site which was also called peripheral anionic site. Simultaneous binding to the catalytic anionic subsite and the peripherical anionic site is responsible for the enhanced binding of gorge-spanning ligands, such as galanthamine and donepezil (E2020). Recent biochemical studies have shown that the peripherical anionic site is also implicated in promoting aggregation of the beta-amyloid (Aβ) peptide responsible for the neurodegenerative process in AD ( Greenblatt et al. 1999 ; Tariot 2001 ; Bar-On et al. 2002 ). Thus, the design of inhibitors interacting with the peripherical anionic site is of clear potential interest for treatment of AD ( Zeng et al. 1999 ).

Bioprospecting is a term coined recently to refer to the search for novel products or microorganisms of economic importance from the world’s biota. Over the past few years a renewed interest has been generated on the investigation of fungi as a potential source of novel bioactive compounds like immunosuppressive, anti-cholesterolemic and anti-tumour agents ( Harvey 2000 ; Strobel 2000 ; Tulp and Bohlin 2004 ). Most fungi investigated have been isolated from soil samples and they have provided a broad spectrum of secondary metabolites with diverse chemical structures; the most exciting recent discoveries have come from exploration of fungi surviving in unusual ecological niches like rain forests ( Strobel 2000 ), marine sponges ( Höller et al. 2000 ), and mangroves ( Lin et al. 2001 sp. FO 4259 (Kuno et al. ). In particular, AChE inhibitors were reported to be produced by some soil fungi like arisugacin A and B from *Penicillium*^1996^ (Kim et al. ), quinolactacins A1 and A2 from *Penicillium citrinum*^2001^ (Kim et al. ) and terreulactones A, B, C and D from *Aspergillus terreus*^2003^ ). The present study reports on the isolation and characterization of fungal acetylcholinesterase (AChE) inhibitors from the hitherto unexplored biosphere, the Kaziranga National Park, Assam, India. The Kaziranga National Park (26° 30′ to 26° 45′N and 93° 05′ to 93° 40′E), Assam, India is located on the southern bank of the Brahmaputra river at the foot of the Mikir – Kirbi Anglang Hills and is declared as a World Heritage site by UNESCO in 1985. This park is one of the last areas in Eastern India and is almost undisturbed by human and has a unique natural riverine landscape of sheer forest, tall elephant grass, rugged reeds, marshes and shallow pools inhabited by the world’s largest population of one-horned rhinoceroses and diverse range of other wild animals ( UNEP 2005 ; UNESCO 2010 ). Considering the importance of this hitherto unexplored biosphere, fungi were isolated from different soil and dung samples collected from this forest and the metabolic profiling of secondary metabolites produced by a newly isolated *Chrysosporium lobatum* strain BK-3 was carried out and assessed for their AChE inhibition, antimicrobial, cytotoxicity and antioxidant activities.

## Materials and methods

### Chemicals used

Acetylcholinesterase (AChE) from electric eel (EC 3.1.1.7); acetylthiocholine iodide (ATCI); 5,5′-dithiobis-(2-nitrobenzoic acid) (DTNB); Trizma hydrochloride (Tris–HCl) buffer solution, pH 8.0 and reference compound like galanthamine hydrobromide from *Lycoris* sp. were purchased from Sigma (St. Louis, MO, USA).

### Fungal strain and fermentation conditions

The fungal strain BK-3 was isolated from a forest soil sample collected from the Kaziranga National Park, Assam, India, as part of a bioactives screening program ( Kumar et al. 2010 ). The pure culture was maintained in the in-house culture repository of the institute with the accession number ICTF-039 and based on the cultural and morphological characteristics of spore and hyphae by lactophenol blue staining and microscopic observation, the strain BK-3 was identified as *Chrysosporium lobatum*. The strain BK-3 was pre-cultured aerobically at 30°C for 5 days in 1 L Erlenmeyer flasks containing 250 ml of potato dextrose broth medium and agitated at 150 rev min^-1^ in an orbital shaker for 120 h. The fermented medium was later filtered through a muslin cheese cloth to remove the fungal biomass and then the infiltrate was centrifuged at 2000 rpm to obtain a cell-free supernatant.

### Extraction, analysis and purification of bioactive metabolites

Bioactive compounds were extracted from the cell-free supernatant by absorption onto Diaion HP-20 (3%, Supelco, Bellafonte, PA, USA) resin. The resin was washed with water and then extracted with methanol to obtain the crude extract. The crude extract was analyzed by thin-layer chromatography (TLC) on silica gel 60 plates (F_254_, Merck). Plates were developed in a methanol-chloroform (5:95) solvent mixture and visualized under UV light at 254 nm which revealed the presence of two bioactive compounds. The extracted compound was further concentrated under reduced pressure on a rotary vacuum evaporator (Rotavapor R-205, Büchi, Switzerland) and further profiled on silica gel (60–120 mesh) column (3 × 60 cm) with methanol-chloroform solvent system as a mobile phase. Spot 1 was eluted in methanol-chloroform mixture (1:99, v/v). The same solvent mixture was continued till compound 1 was completely eluted and after drying gave a pale yellow powder. Spot 2 was eluted in methanol-chloroform mixture (1.5:98.5, v/v), and drying of the fractions resulted in compound 2 as white powder. Both compounds were UV-active and visualized under UV light at 254 nm, as well as by spraying with phosphomolybdic acid followed by heating at 100°C for 2–3 min which appeared as blue coloured spots.

### Structural characterization

The purified compounds were further subjected to ^1^H and ^13^C NMR, FT-IR, ESI-MS along with 2D-NMR spectroscopic studies like HMBC, HSQC, ^1^H-^1^H-COSY, NOSEY and DEPT-135 studies for elucidating their structures. The UV and visible spectra were measured by dissolving the samples in spectroscopic acetonitrile and recorded at 30°C on a UV-visible double beam spectrophotometer (Lambda 25, Perkin-Elmer, Shelton, CT). Nuclear magnetic resonance (NMR) spectra were recorded on a Bruker Avance 300 MHz NMR spectrometer (Bruker, Switzerland). The ^1^H and ^13^C NMR spectra were determined in deuterated chloroform at room temperature, and chemical shifts were represented in *δ* values (ppm) with tetramethylsilane (TMS) as an internal standard. The Fourier transform infrared spectrum (FT-IR) was performed using the Thermo-Nicolet Nexus 670 FT-IR spectrophotometer (Thermo Fisher Scientific Inc., Madison, WI, USA) using KBr pellets and spectra were collected at a resolution of 4 cm^-1^ in the wavenumber region of 400–4,000 cm^-1^. The high resolution mass spectra (HR-MS) were recorded on a QSTAR XL Hybrid ESI-Q TOF mass spectrometer (Applied Biosystems Inc., Fosters City, CA, USA). Electrothermal Digital 9000 Series melting point apparatus (Model IA9200, Barnstead, UK) was used for determining the melting point of the purified compounds. The purified sample (1 mg) of each bioactive compound was placed in a glass capillary tube and this tube was placed in an aluminium heating block which was heated at a fast ramp rate of 10°C min^-1^ from a temperature range of 25 to 300°C for melting point measurement.

### Antimicrobial activity

Antimicrobial activity was determined using the broth dilution method as described previously ( Kumar and Mamidyala 2011 ). The target strains used for screening the antimicrobial activities were *Micrococcus luteus* MTCC 2470, *Staphylococcus aureus* MTCC 96, *Staphylococcus aureus* MLS16 MTCC 2940, *Bacillus subtilis* MTCC 121, *Escherichia coli* MTCC 739, *Pseudomonas aeruginosa* MTCC 2453, *Klebsiella planticola* MTCC 530 and *Candida albicans* MTCC 3017. These strains were procured from Microbial Type Culture Collection and Gene Bank (CSIR-Institute of Microbial Technology, Chandigarh, India).

### *In vitro* cytotoxicity testing

Cytotoxicity of the compounds was assessed on the basis of the measurement of the *in vitro* growth in the 96 well plates by cell-mediated reduction of tetrazolium salt to water insoluble formazan crystals by a previously described method ( Mosmann 1983 ). Cell lines for testing *in vitro* cytotoxicity included HeLa derived from human cervical cancer cells (ATCC No. CCL-2), A549 derived from human alveolar adenocarcinoma epithelial cells (ATCC No. CCL-185), MDA-MB-231 derived from human breast adenocarcinoma cells (ATCC No. HTB-26), MCF7 derived from human breast adenocarcinoma cells (ATCC No. HTB-22), COLO 205 derived from human colon cancer cell line (ATCC No CCL-222), K562 derived from human chronic myelogenous leukemia cell line (ATCC No. CCL-243) and HEK 293 derived from human embryonic kidney cell line (ATCC No. CRL-1573) were obtained from American Type Culture Collection, Manassas, VA, USA. The IC_50_ values (50% inhibitory concentration) were calculated from the plotted absorbance data for the dose–response curves. The IC_50_ values (in μM) were expressed as the average of two independent experiments.

### Antioxidant activities

The purified compounds were investigated for their antioxidant potentials in different oxidizing systems, viz., radical scavenging activity by 1,1-diphenyl-2-picrylhydrazyl (DPPH) reduction ( Kumar et al. 2011 ), superoxide radical scavenging activity ( Liu et al. 1997 ), inhibition of lipid peroxidation ( Zhang and Yu 1997 ) and hemolysis in erythrocyte membrane stabilization ( Ng et al. 2000 ) at different concentrations. Ascorbic acid and luteolin were also employed as standards in these assays. All experiments were carried out in triplicates and the EC_50_ (50% effective concentration) values (μg ml^-1^) were expressed as the mean ± standard deviation (S.D.).

### In vitro acetylcholinesterase (AChE) assay

AChE inhibitory activity for the purified compounds was performed using the modified method of Ellman et al ( 1961 ) in 96 well plates ( Rhee et al. 2001 ). The concentration of the compound that inhibited 50% of AChE activity (IC_50_) was estimated by plotting percent activity and percent inhibition of AChE versus inhibitor concentrations on the same graph. The concentration at the intersection of these two curves was the IC_50_ value.

### Protein–ligand docking studies

The molecular docking studies were performed using various docking softwares like GOLD (Genetic Optimization for Ligand Docking, version 3.2) and CDOCKER (in Discovery Studio 2.5) on the curvularin molecule with the well-known complex acetylcholinesterase complexed with E2020 which is marketed as Aricept (PDBID: 1EVE) ( Badrinarayan et al. 2011 ). The Accelrys Discovery studio protein preparation wizard was used to prepare the protein with default settings. The ligands were subjected to energy minimization at AM1 level of theory. The default parameters in GOLD 3.2 such as number of islands 5, population size of 100, number of operations was 100,000, a niche size of 2, a selection pressure of 1, the van der Waals and hydrogen bonding were set to 4.0 and 2.5, respectively, have been used to perform GOLD docking calculations ( Srivani et al. 2007 ; Ravindra et al. 2008 ; Badrinarayan and Sastry 2011 ). The CDOCKER had an all-atom CHARMm force field-based docking algorithm and used the soft-core potentials with an optional grid representation to dock ligands into the active site of the receptor. CDOCKER generated the random ligand conformations through molecular dynamics simulation. CDOCKER docking was done with the default parameters using the ligand E2020 as the center.

## Results

### Purification, characterization and structure elucidation of bioactive compounds

**Figure 1:**
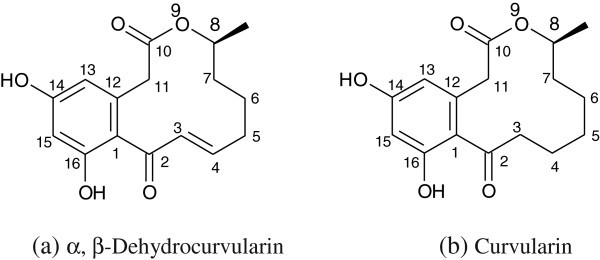
**Structures for (a) α, β-dehydrocurvularin and (b) curvularin produced by*Chrysosporium lobatum*strain BK-3.**

The culture filtrate of strain BK-3 revealed the presence of two major spots in TLC with *Rf* values of 0.5 for compound 1 and 0.3 for compound 2 which were separated in a solvent mixture of methanol:chloroform solvent system. *Compound 1*: Yield: 50 mg l^-1^; pale yellow powder; heat stable and showed no change in its activity, when autoclaved at 121°C for 30 min; mp 218–220°C. UV λ_max_ (EtOH) (log_e_): 225 (4.247). ^1^H-NMR (DMSO-d_6_ 500 MHz) δ (ppm): 1.11 (3H, d, J = 6.42, 4-CH3), 1.46 (1H, m, H-6), 1.48 (1H, m, H-5), 1.75 (1H, m, H-5), 1.82 (1H, m, H-6), 2.19 (1H, m, H-7), 2.28 (1H, m, H-7), 3.36 (1H, d, J = 15.57, H-1), 3.39 (1H, d, J = 15.57, H-1), 4.74 (1H, m, H-4), 6.23 (2H, s, H-12, H-14), 6.3 (1H, d, J = 15.74, H-9), 6.38 (1H, m, H-8). ^13^C-NMR (DMSO-d_6_ 500 MHz) δ (ppm): 20.11 (q, 4-CH_3_), 23.94 (t, C-6), 32.9 (t, C- 7), 33.53 (t, C-5), 39.94 (t, C-1), 72.28 (d, C-4), 101.62 (d, C-12), 109.71 (d, C-14), 118.18 (s, C-16), 132.72 (d, C-9), 133.95 (s, C-15), 153.75 (d, C-8), 157.62 (s, C-13), 159.25 (s, C-11), 170.43 (s, C-2), 197.75 (s, C-10). IR (KBr) (ν_max_ cm^-1^): 3470 and 3300 (hydroxyl), 1720 (lactone), 1600 (conjugated aromatic system), 1300 and 970 (conjugated trans-CH = CH-), 843 (1,2,3,5-tetrasubstituted benzene). MS (ESI) *m/z*: 291.1 [M + H]^+^; HR-MS (ESI) *m/z* calculated for C_16_H_18_O_5_ [M + H]^+^: 290.32, found: 291.122. Based on the spectral data (Additional file 1: Figure S1-S7) and its comparison with that of the authentic data from the reported literature ( Munro et al. 1976 ; Arai et al. 1989 ; Jiang et al. 2008 ), the compound 1 was identified as α, β-dehydrocurvularin (Figure 1).

*Compound 2*: Yield: 60 mg l^-1^; white powder (MeOH), mp 205–208°C. UV λ_max_ (EtOH) (log_e_): 225 (4.247). MF: C_16_H_20_O_5_; ^1^H NMR (CDCl_3_, 300 MHz) δ (ppm): 9.49 (1 H, s, 16-OH), 9.26(1 H, s, 14-OH), 6.38 (1 H, s, H-15), 6.15 (1 H, d, H-13), 4.87 (1 H, m, H-8), 3.64 (2 H, s, H-11), 2.77 (1 H, m, H-3a), 2.56 (1 H, d, m, H-3b), 1.77-1.18 (8 H, m, H-4,5,6,7), 1.14 (3 H, d, 8-CH3).^13^C NMR data (CDCl_3_, 300 MHz) δ (ppm): 206.05 (C-2), 170.19 (C-10), 159.13 (C-16), 157.28 (C-14), 135.28 (C-12), 119.62 (C-1), 110.92 (C-13), 101.59 (C-15), 71.42 (C-8), 42.89 (C-3), 40.33 (C-11), 31.56 (C-7), 26.18 (C-5), 23.28 (C-6), 22.26 (C-4), 20.08 (8-CH3). IR (KBr): 3425 (br), 2924, 1722, 1609, 1592, 1264. MS (ESI) *m/z*: 293 [M + H]^+^; HR-MS (ESI) *m/z* calculated for C_16_H_20_O_5_ [M + H]^+^: 292.33, found: 292.11. The spectral data of the compound 2 (Additional file 1: Figure S8; Additional file 1: Figure S9; Additional file 1: Figure S10; Additional file 1: Figure S11; Additional file 1: Figure S12; Additional file 1: Figure S13;Additional file 1: Figure S14; Additional file 1: Figure S15; Additional file 1: Figure S16) were identical with that of the authentic data from the reported literature ( Musgrave 1957 ; Ghisalberti et al. 1993 ) and was identified as curvularin (Figure 1).

### Acetylcholine esterase inhibitory activity

The initial crude extract showed 60% of AChE inhibitory activity. Among the two purified compounds, only curvularin exhibited positive AChE inhibitory activity showing 80% inhibition as compared to standard galanthamine which showed 75% inhibition. The IC_50_ values determined for curvularin and galanthamine (standard) were 1.36 and 2.625 μM ml^-1^.

### Antimicrobial susceptibility testing and cytotoxicity

Antimicrobial susceptibility tests of the purified metabolites profiled from strain BK-3 indicated that both the compounds did not exhibit any antimicrobial activity against all the tested Gram-positive and -negative bacteria and *Candida albicans*. Further, the *in vitro* cytotoxicity evaluated against a panel of human tumor cell lines (Table 1), indicated that both curvularin and α, β-dehydrocurvularin had similar level of anti-tumour activity against A549, HeLa, MDA-MB-231 and MCF-7, while α, β-dehydrocurvularin was active against COLO 205 with an IC_50_ of 7.9 μM; however, curvularin was inactive.

**Table 1:** ***In vitro*cytotoxicity of bioactive compounds produced by*Chrysosporium lobatum*strain BK-3**

Compounds	IC_50_values (μM) (Mean ± S.D.^b^)						
	MCF-7	A549	MDA-MB-231	COLO 205	K562	HeLa	HEK293 (normal)
α, β-Dehydrocurvularin	11.19 ± 0.31	2.10 ± 0.33	9.34 ± 0.38	7.9 ± 0.09	–^a^	21.01 ± 0.19	–
Curvularin	21.89 ± 0.19	13.91 ± 0.15	1.3 ± 0.37	–	–	25.64 ± 0.37	–
Doxorubicin (Standard)	1.05 ± 0.37	1.21 ± 0.41	0.501 ± 0.26	0.985 ± 0.33	0.318 ± 0.26	0.451 ± 0.25	–

^a^– No activity; ^b^S.D.: Standard deviation.

### Antioxidant activities

The EC_50_ (μg ml^-1^) values of the total DPPH scavenging, superoxide anion scavenging, lipid peroxidation inhibition, erythrocyte hemolytic inhibition activities of curvularin, α, β-dehydrocurvularin, ascorbic acid and luteolin (as standards) is shown in Table 2. α, β-Dehydrocurvularin showed significant antioxidant potential, however, curvularin did not exhibit any antioxidant activity.

**Table 2:** **Antioxidant properties of bioactive compounds produced by*Chrysosporium lobatum*strain BK-3**

Assays	EC_50_(μg ml^-1^) (Mean ± S.D.^b^)			
	α, β–Dehydrocurvularin	Curvularin	Ascorbic acid (Standard)	Luteolin (Standard)
DPPH scavenging activity	50.75 ± 0.14	–^a^	40.28 ± 0.27	44.18 ± 0.15
Superoxide anion scavenging activity	16.71 ± 0.16	–	21.01 ± 0.36	31.01 ± 0.11
Lipid peroxidation inhibition	159.09 ± 0.09	–	139.97 ± 0.30	134.1 ± 0.19
Erythrocyte hemolytic inhibition	141.79 ± 0.21	–	119.81 ± 0.30	122.00 ± 0.10

^a^– No activity; ^b^S.D.: Standard deviation.

### Molecular docking studies

**Figure 2:**
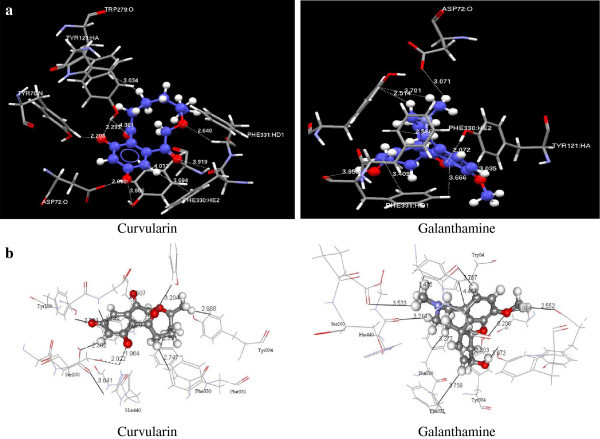
**The typical residue interactions of curvularin and galanthamine in the active site of acetylcholinesterase enzyme (PDBID: 1EVE) using (a) GOLD and (b) CDOCKER docking protocols.**

The molecular docking studies were carried out to evaluate the binding affinity of curvularin and the reversible cholinesterase inhibitor galanthamine with acetyl cholinesterase (AChE) enzyme using GOLD (version 3.2) and CDOCKER (Discovery Studio version 2.5) docking softwares. These studies were performed on curvularin and galanthamine with well-known enzyme acetylcholinesterase complexed with E2020 which is marketed as Aricept (PDBID: 1EVE) ( Kryger et al. 1999 ). The active site radius was set to 10 Å, which formed the atom number 1284, and the nitrogen atom of the active site residue Trp84, which formed π–π interactions with E2020. The default parameters had been used for the docking studies in the above mentioned docking softwares. The GOLD docking results showed good activity with 49.736 and 52.06 docking scores, CDOCKER showed 53.93 and 37.72 docking scores for curvalarin and galanthamine, respectively (Table 3). The interactions of ligands in the binding site have been depicted in Figure 2.

**Table 3:** **Docking results of curvularin produced by*Chrysosporium lobatum*strain BK-3 with acetyl cholinesterase enzyme (PDBID: 1EVE) using various docking protocols**

Structure	Different docking protocols	
	GOLD	CDOCKER
Curvularin	44.40	53.93
Galanthamine	52.06	37.72

The compound curvularin exhibited strong hydrogen bonding interactions (CH-O and OH-O) with ASP72, GLY119 and PHE331 residues. The N-H of the HIS440 residue showed a strong hydrogen bonding interaction with oxygen of the hydroxyl group presented in the curvularin. The ligand formed OH-O type of hydrogen bonding interactions with hydroxyl groups of tyrosine residues TYR334, 70 and 121 in the active site. The PHE330, 331 and TYR334 residues formed hydrophobic interactions with the curvularin ligand. All the above mentioned interactions were in the range of less than 4.00 Å between ligand and binding site residues. The curvularin showed similar interactions as Aricept ligand, the bottom of the binding site showed a strong interaction on one face of the benzyl ring and displayed a classic π–π stacking with the six-membered ring of TRP84 indole. At the middle of the binding site curvularin showed an interaction with PHE330 and TYR121 residues in the hydrophobic fashion. On other hand this ligand also formed a strong hydrogen bond interaction with HIS440 residue. The docking results of curvularin molecule were compared with the existing drug galanthamine to compare the various parameters such as score and different type of interactions. Galanthamine showed strong hydrogen bonding interactions with ASP72, GLY118 and TYR121 residues similar to curvularin, however, the strong hydrogen bonding interactions with HIS440 and GLY119 residues which was absent in galanthamine. The galanthamine showed another important hydrophobic interaction with PHE331 and TYR334 which is similar to curvularin interactions in the middle of the binding site. The above analysis clearly shows that curvularin molecule showed similar type of interactions as the existing drug galanthamine in the active site.

## Discussion

AChE inhibitors have therapeutic applications in AD, senile dementia, ataxia, myasthenia gravis and Parkinson’s disease ( McGleenon et al. 1999 ). Global interests in natural products have increased of late due to safety concerns of synthetic products. All of the known AChE inhibiting drugs used in the clinics suffer from several side effects such as high toxicity, short duration of biological action, low bioavailability and narrow therapeutic effects ( Lee et al. 2011 sp. (Kuno et al. ). Consequently, there is a need for the development of new AChE inhibitors with less toxicity and more potency. Only three fungal species like *Penicillium*^1996^ ; Kim et al. 2001 (Kim et al. ), *Aspergillus terreus*^2003^ sp. (Wen et al. ), and *Sporothrix*^2009^ ) have been reported so far to produce compounds with AChE inhibitory activity. In the present study, we report the bioassay-guided purification of α, β-dehydrocurvularin and curvularin (Figure 1) from *Chrysosporium lobatum* (Caputo and Viola strain BK-3 and their structure elucidation. Based on the AChE inhibition and cytotoxicity screening assays, we found that curvularin and α, β-dehydrocurvularin have slightly different bioactivity profiles. α, β-Dehydrocurvularin and curvularin and their derivatives were earlier reported to be produced by some fungi such as *Aspergillus aureofulgens*^1977^ (Arai et al. ), *Alternaria cinerariae*^1989^ (Bicalho et al. ), *Curvularia eragrostidis*^2003^ sp. (Zhan et al. ), *Penicillium*^2004^ (Gutierrez et al. ), *Nectria galligena*^2005^ sp. (Xie et al. ) and *Eupenicillium*^2009^ ). Curvularin is a macrolide exhibiting antibiotic activity towards some fungi and acted as a non-specific phytotoxin ( Robeson and Strobel 1981 (Kusano et al. ). Other activities reported for curvularin and its derivatives are as potential nematicides against the root-lesion nematode, *Pratylenchus penetrans*^2003^ ) and as a bioherbicide ( Jiang et al. 2008 ). More interestingly, these derivatives were identified as cell division inhibitors ( Ghisalberti et al. 1993 ), specifically disordering the microtubule centers ( Almassi et al. 1994 ) and inducing barrel-shaped spindles ( Kobayashi et al. 1988 ).

The anti-tumour activity of curvularin was not reported earlier and the IC_50_ values were assayed according to the MTT method. The results showed that both curvularin and α, β-dehydrocurvularin were active against the tumour cell lines. The IC_50_ values of α, β-dehydrocurvularin were almost always one order of magnitude higher than those of curvularin against the corresponding test cell lines except for MDA-MB-231 cell line, which might be due to the lack of the 3,4-double bond (unsaturation) in curvularin. Based on their chemical structures (Figure 1), there is only one functional group difference between the two compounds; α, β-dehydrocurvularin has a double bond between C-3 and C-4 position, while curvularin has none in the corresponding position. In case of flavonoid-type compounds, the structure-activity relationship (SAR) was explained by the fact that the presence of a double bond is the only structural difference between two flavonoids (as one counterpart), which is one of the important structural property essential for effective interaction between flavonoid and the breast cancer resistance protein, and the flavonoid compound with double bond is more active than its counterpart ( Zhang et al. 2005 ). It was earlier reported that the bioactivity of some other class of chemical compounds with double bond was notably stronger than those of their corresponding hydrogenated derivatives ( Dizhbite et al. 2004 ; González-Díaz et al. 2007 ; Cheng et al. 2008 ). In one report ( Gutierrez et al. 2005 ), it was indicated that α, β-dehydrocurvularin produced by *Nectria galligena* showed no IC_50_ value reported for α, β-dehydrocurvularin even at the use concentration of >300 μm. Further, the α, β-dehydrocurvularin exhibited cytotoxic activity with IC_50_ value of <12 μg ml^-1^ against human lung fibroblast cell line (MRC-5), while the α, β-dehydrocurvularin produced by *Chrysosporium lobatum* strain BK-3 showed cytotoxic activity with IC_50_ values of 2.10 μM against A549, 7.9 μM against COLO-205 and 9.34 μM against MDA-MB-231. From these results, it clears that α, β-dehydrocurvularin from *Nectria galligena* exhibited only cytotoxic activity but no AChE inhibitory activity. Further, α, β-dehydrocurvularin from *Nectria galligena* exhibited antimicrobial activity only against *Pseudomonas syringae* with IC_350_ value of 14.2 μg ml^-1^, while α, β-dehydrocurvularin from *Chrysosporium lobatum* did not exhibit any antimicrobial activity against the tested microbial strains.

## Conclusions

This study demonstrated that curvularin and α, β-dehydrocurvularin were the two main structurally related secondary metabolites produced by *Chrysosporium lobatum* strain BK-3 with slightly different bioactive profiles. Both these compounds were active against different cancer cell lines. However, α, β-dehydrocurvularin did not exhibit inhibition for AChE, while curvularin did, which suggested that the partial planar backbone structure in the presence of a 3,4-double bond played an important role in the inhibition of AChE activity. Further, the docking analysis showed that similar to Aricept ligand, curvularin showed a strong hydrogen bonding and hydrophobic interactions in the bottom and middle of the binding site of AChE enzyme. Curvularin had good docking scores as compared to the existing drug galanthamine; in case of CDOCKER curvularin had higher docking score than galanthamine (53.931 and 37.72, respectively). The docking study clearly revealed that the curvularin molecule had better interactions and it was fitting very well into the receptor of AChE enzyme. It was showing better interactions than galanthamine. To the best of our knowledge this is a first study on the characterization of metabolites of *Chrysosporium lobatum* strain BK-3 isolated from Kaziranga National Park biosphere exhibiting AChE inhibitory, cytotoxicity and antioxidant activities.

## Electronic supplementary material

Additional file 1: Figure S1: ^1^H NMR spectrum of α, β-dehydrocurvularin. **Figure S2.**^13^C NMR spectrum of α, β-dehydrocurvularin. **Figure S3.** FT-IR spectrum of α, β-dehydrocurvularin. **Figure S4.** HR-MS spectrum of α, β-dehydrocurvularin. **Figure S5a.** HMBC spectrum (A) and its expansion (B) of α, β-dehydrocurvularin. **Figure S5b.** HMBC spectrum and its expansions (C and D) of α, β-dehydrocurvularin. **Figure S6a.** HSQC spectrum (A) of α, β-dehydrocurvularin. **Figure S6b.** HSQC spectrum and its expansions (B and C) of α, β-dehydrocurvularin. **Figure S7.** DEPT-135 spectrum of α, β-dehydrocurvularin. **Figure S8.**^1^H NMR spectrum of curvularin. **Figure S9.**^13^C NMR spectrum of curvularin. **Figure S10.** FT-IR spectrum of curvularin. **Figure S11.** HR-MS spectrum of curvularin. **Figure S12a.**^1^H-^1^H COSY spectrum (A) of curvularin. **Figure S12b.**^1^H-^1^H COSY spectrum and its expansions (B and C) of curvularin. **Figure S13.** NOESY spectrum (A) and its expansion (B) of curvularin. **Figure S14a.** HMBC spectrum (A) of curvularin. **Figure S14b.** HMBC spectrum and its expansions (B and C) of curvularin. **Figure S15a.** HSQC spectrum (A) of curvularin. **Figure S15b.** HSQC spectrum and its expansions (B and C) of curvularin. **Figure S16.** DEPT-135 spectrum of curvularin. (DOC 3 MB)
