# Supplementary material for: Metabolite profiling and biological activities of bioactive compounds produced by Chrysosporium lobatum strain BK-3 isolated from Kaziranga National Park, Assam, India
Source: Springerplus. 2013 Mar 21;2:122. doi: 10.1186/2193-1801-2-122 (PMC3616213; doi:10.1186/2193-1801-2-122)
Supplement: Supplementary file 1 — Additional file 1: Figure S1: 1H NMR spectrum of α, β-dehydrocurvularin. Figure S2.13C NMR spectrum of α, β-dehydrocurvularin. Figure S3. FT-IR spectrum of α, β-dehydrocurvularin. Figure S4. HR-MS spectrum of α, β-dehydrocurvularin. Figure S5a. HMBC spectrum (A) and its expansion (B) of α, β-dehydrocurvularin. Figure S5b. HMBC spectrum and its expansions (C and D) of α, β-dehydrocurvularin. Figure S6a. HSQC spectrum (A) of α, β-dehydrocurvularin. Figure S6b. HSQC spectrum and its expansions (B and C) of α, β-dehydrocurvularin. Figure S7. DEPT-135 spectrum of α, β-dehydrocurvularin. Figure S8.1H NMR spectrum of curvularin. Figure S9.13C NMR spectrum of curvularin. Figure S10. FT-IR spectrum of curvularin. Figure S11. HR-MS spectrum of curvularin. Figure S12a.1H-1H COSY spectrum (A) of curvularin. Figure S12b.1H-1H COSY spectrum and its expansions (B and C) of curvularin. Figure S13. NOESY spectrum (A) and its expansion (B) of curvularin. Figure S14a. HMBC spectrum (A) of curvularin. Figure S14b. HMBC spectrum and its expansions (B and C) of curvularin. Figure S15a. HSQC spectrum (A) of curvularin. Figure S15b. HSQC spectrum and its expansions (B and C) of curvularin. Figure S16. DEPT-135 spectrum of curvularin. (DOC 3 MB) [file 40064_2012_187_MOESM1_ESM.doc]

**Supplementary data**

**Metabolite profiling and biological activities of bioactive compounds produced by *Chrysosporium lobatum* strain BK-3 isolated from Kaziranga National Park, Assam, India**

C. Ganesh Kumar^a^,*, Poornima Mongolla^a^, Pombala Sujitha^a^, Joveeta Joseph^a^, K. Suresh Babu^b^, Gangi Suresh^b^, K.V.S. Ramakrishna^c^, Uppula Purushotham^d^, G. Narahari Sastry^d^ and Ahmed Kamal^a^

^a^Chemical Biology Laboratory,

^b^Natural Products Chemistry Division,

^c^Nuclear Magnetic Resonance Centre,

^d^Molecular Modelling Group,

CSIR-Indian Institute of Chemical Technology, Uppal Road, Hyderabad 500 607, India

**Corresponding author*

Mailing address: Chemical Biology Laboratory, CSIR-Indian Institute of Chemical Technology, Uppal Road, Hyderabad 500607, Andhra Pradesh, India

Phone: +91-40-27193105; Fax: +91-40-27193189.

Email address: [cgkumar@iict.res.in](mailto:cgkumar@iict.res.in); [cgkumar1@rediffmail.com](mailto:cgkumar1@rediffmail.com)

**Figure legends for Supplementary data**

Supplementary Figure S1. ^1^H NMR spectrum of αβ-dehydrocurvularin

Supplementary Figure S2. ^13^C NMR spectrum of αβ-dehydrocurvularin

Supplementary Figure S3. FT-IR spectrum of αβ-dehydrocurvularin

Supplementary Figure S4. HR-MS spectrum of αβ-dehydrocurvularin

Supplementary Figure S5a. HMBC spectrum (A) and its expansion (B) of αβ-dehydrocurvularin

Supplementary Figure S5b. HMBC spectrum and its expansions (C and D) of αβ-dehydrocurvularin

Supplementary Figure S6a. HSQC spectrum (A) of αβ-dehydrocurvularin

Supplementary Figure S6b. HSQC spectrum and its expansions (B and C) of αβ-dehydrocurvularin

Supplementary Figure S7. DEPT-135 spectrum of αβ-dehydrocurvularin

Supplementary Figure S8. ^1^H NMR spectrum of curvularin

Supplementary Figure S9. ^13^C NMR spectrum of curvularin

Supplementary Figure S10. FT-IR spectrum of curvularin

Supplementary Figure S11. HR-MS spectrum of curvularin

Supplementary Figure S12a. ^1^H-^1^H COSY spectrum (A) of curvularin

Supplementary Figure S12b. ^1^H-^1^H COSY spectrum and its expansions (B and C) of curvularin

Supplementary Figure S13. NOESY spectrum (A) and its expansion (B) of curvularin

Supplementary Figure S14a. HMBC spectrum (A) of curvularin

Supplementary Figure S14b. HMBC spectrum and its expansions (B and C) of curvularin

Supplementary Figure S15a. HSQC spectrum (A) of curvularin

Supplementary Figure S15b. HSQC spectrum and its expansions (B and C) of curvularin

Supplementary Figure S16. DEPT-135 spectrum of curvularin

Supplementary Figure S1

Supplementary Figure S2

Figure S3


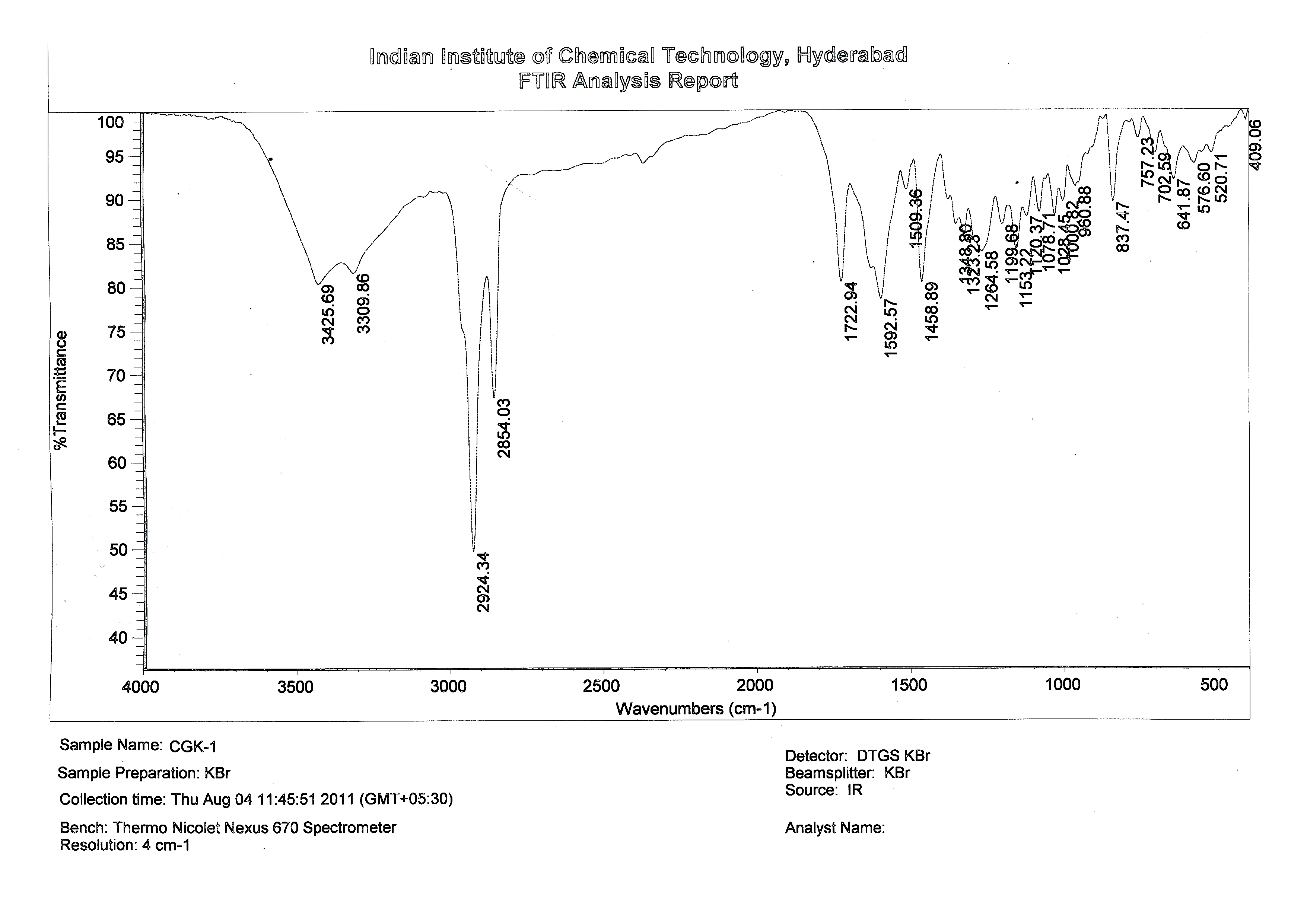


**% Transmittance**

**Wavenumbers (cm^-1^)**

Supplementary Figure S4

**Intensity (counts)**

***m/z* (amu)**

Supplementary Figure S5a

**A**

**B**

Supplementary Figure S5b

**C**

**D**

Supplementary Figure S6a

**A**

Supplementary Figure S6b

**B**

**C**

Supplementary Figure S7

Supplementary Figure S8

Supplementary Figure S9

Supplementary Figure S10


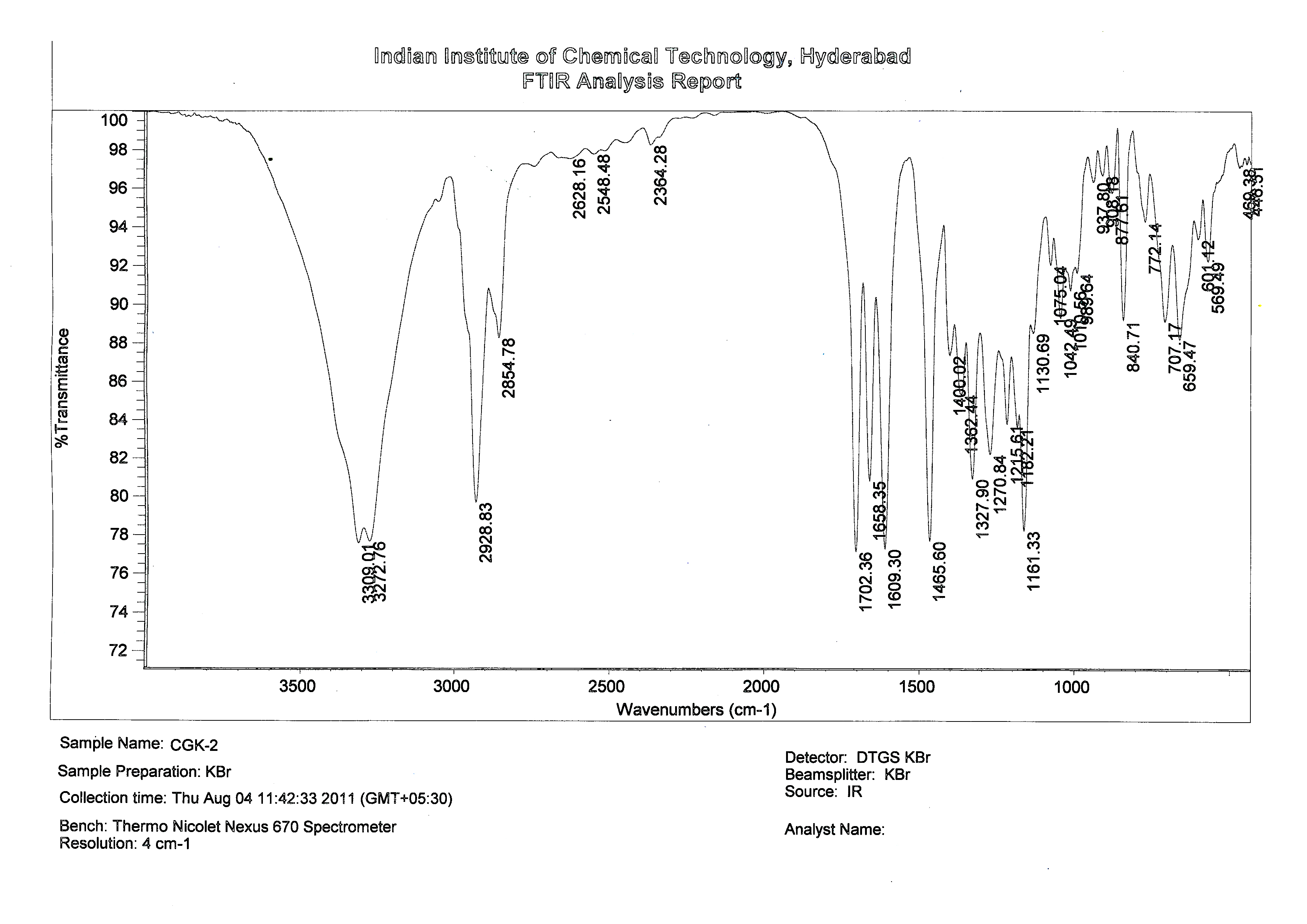


**% Transmittance**

**Wavenumber (cm^-1^)**

Supplementary Figure S11

***m/z* (amu)**

**Intensity (counts)**

Supplementary Figure S12a

**A**

**A**

Supplementary Figure S12b

**B**

**C**

Supplementary Figure S13

**B**

**A**

Supplementary Figure S14a

**A**

Supplementary Figure S14b

**B**

**C**

Supplementary Figure S15a

**A**

Supplementary Figure S15b

**B**

**C**

Supplementary Figure S16
